# Personal News Items

**Published:** 1915-02

**Authors:** 


### Personal News Items.

Dr. P. F. Robertson of Stockdale has located in Devine.

Dr. W. O. Brown of North Texas will reside permanently in Beeville.

Dr. Eugene Baliss has moved from Richards and is now located in Huntsville, Texas.

Dr. H. Allison of Kingsville has the sympathy of his many friends in the loss of his father.

Dr. L. D. Wood has moved from Cooper to Enloe, where he has established himself permanently.

Dr. A. W. Brown of Richland has been on the sick list for some time but is reported much better.

Dr. Hamil of Dallas has located in Richardson to practice medicine and to make his home there.

Dr. and Mrs. W. C. Mayes of Memphis have returned from a visit with relatives at Alexandria, La.

Dr. Thurston of Beeville, who has been quite ill, is gradually improving and will soon be well again.

Dr. E. U. Wood has moved from Judsonia, Ark., to Sabinal, Texas, where he will open his office soon.

Dr. J. M. Townes and Dr. J. T. Sellman of Joshua have both been quite ill, but are able to be up again.

Dr. Hamill of Dallas has concluded to locate permanently at Handley, where he resided a few years ago.

Dr. J. E. Hall of Kiamichi, Okla., has moved to Woodlawn, Texas, where he will make his future home.

Dr. J. G. Davis and wife of Weston have moved to Point, Texas, where Dr. Davis will practice his profession.

Dr. R. E. Burrus has moved from Mt. Pleasant to Winfield, where he expects to practive the coming year.

The people of Eldorado are welcoming most cordially Dr. R. H. Greer of Dallas, who has gone to Eldorado to practice.

The friends of Dr. and Mrs. W. H. Henthorne of Roscoe are expressing regret at their removal to Colorado, Texas.

Dr. L. W. Hollis of Abilene has been quite ill with the grippe; he is improving, however, and will soon be about again.

Dr. A. A. Jackson has removed from Kosse to Personville, near Mexia, where he will practice his profession in the future.

Dr. Howard of Bagwell has moved to Baird, Texas.

Dr. H. P. Oliver and family of Farwell, Texas, have moved to Lorenzo, Crosby county, where they will reside in the future.

We sympathize with Dr. and Mrs. Meeks of Collinsville in the loss of their baby boy, whose death occurred on December 27.

Dr. Arnold Denman of Rogers has moved to Belton, where he and his family are finding a glad welcome from his boyhood friends.

Dr. P. H. Harrell of Martindale has associated himself with Dr. A. B. Parr of Gonzales in the practice of medicine and surgery.

Dr. J. W. Kuykendall and son of Fort Worth, Texas, were seriously injured when the street car and the doctor's machine collided.

Dr. R. B. Longmire has moved from Glenfawn, Rusk county, to Jacksonville, where he has formed a partnership with Dr. H. V. Collins.

Dr. and Mrs. W. B. Mackey of Fort Worth announce the arrival of a little daughter on January 17. The young lady weighed seven pounds.

Dr. I. L. McGlasson, for the past four years State quarantine officer at Galveston, will return to his home in Waco and resume his practice.

Dr. J. M. Tribble of Cuero has recently been appointed city health officer of that progressive little town. His friends expect much of him.

Dr. H. F. Pettigrew of Nevada, Texas, has moved to Thurber, Texas, where he has accepted a position with the Texas & Pacific Coal Company.

Dr. H. Kuehne and family of Walburg have moved to Austin. Everybody regretted to see the doctor leave Walburg, as he is a very fine man.

Dr. Cunningham of Ravenna is in Austin to look after the health of the legislators and do what he may to secure good legislation for his people.

Dr. William M. Brumby of Waco was elected president of the Medical Directors' Association, while Dr. M. M. Smith of Dallas was re-elected secretary.

Dr. J. T. Hutchinson of Lubbock left January 6 for Chicago, where he will take a post-graduate course. He will be absent until the first of February.

Dr. W. P. Barron, who far a number of years has been company physician for the Kirby Lumber Company at Carmona, has transferred his seat of action to Rusk, Texas.

Dr. and Mrs. R. B. Anderson entertained the doctors of Seguin on the evening of December 14 most royally. A big supper was prepared by the doctors' wives for the doctors.

Dr. H. L. Warwick of Fort Worth, who has just returned from Europe, talks most interestingly of conditions there. While abroad the doctor did post-graduate work in London, Eng.

Dr. R. G. Powell of Baird is in Austin attending the regular session of the Legislature. He is a member, re-elected, from the One Hundred and Eighth District, Callahan and Eastland counties.

Dr. J. Wooten Smith of San Antonio, who has for many years past been surgeon for the J. M. O'Rourke and U. S. Hospital, has recently moved to Gonzales, where he will in the future make his home.

Dr. Clarence D. Dixon has been appointed president of the board of health of San Antonio to succeed Dr. William M. Brumby, who recently resigned to become chief surgeon for an insurance company in Waco.

Dr. Johnson of Loving has had a real serious time with blood poison in his left hand. He accidentally cut himself with a lance while lancing a rising for a patient. The cut developed blood poison and has caused the doctor much suffering.

Drs. Brown and Knight of Garland have formed a partnership and will office together. Dr. Brown has begun daily work in the medical department of Baylor University at Dallas as assistant pathologist, and Dr. Knight will attend to his office work during his absence.

Dr. Lewis, a prominent physician at Nugent, happened to a painful accident recently when he was caught in a peanut thresher. Both legs and several ribs were broken and he was badly bruised. He was carried to the hospital at Abilene. It is said that there is but little hope of his recovery.

Gonzales has a new professional firm which has just been formed, between Dr. J. A. Maness, Dr. L. Penrod and Dr. G. Schulze for the practice of medicine. The firm name reads, Maness, Schulze & Penrod, physicians and surgeons. Dr. Schulze is from Shiner, where he was connected with Dr. Gray in the management of the Shiner hospital.

Dr. J. H. Traylor, who with his family has been living in Cuero for the past three years has moved to Odessa, Texas, where the doctor will establish a hospital. Dr. Traylor was city health officer during his residence in Cuero and his fearlessness and impartiality endeared him to the people. He will succeed—such men have struck the word "fail" out of their vocabulary.

